# Quantitative Genetics of Smoltification Status at the Time of Seawater Transfer in Atlantic Salmon (*Salmo Salar*)

**DOI:** 10.3389/fgene.2021.696893

**Published:** 2021-11-01

**Authors:** Hooi Ling Khaw, Bjarne Gjerde, Solomon A. Boison, Elise Hjelle, Gareth F. Difford

**Affiliations:** ^1^ Department of Breeding and Genetics, Nofima AS, Osloveien, Norway; ^2^ Mowi ASA, Bergen, Norway; ^3^ PHARMAQ Analytiq AS, Bergen, Norway

**Keywords:** smoltification, atlantic salmon, 0+ and 1+ smolts, heritability, genetic correlations, optimum seawater transfer

## Abstract

High mortality during grow out in the sea is a challenge for farmed Atlantic salmon production in Norway and globally, which is partly attributed to suboptimal smolt quality. In this study, two groups of pre-smolts were put on a standard light smoltification regime with alternating 12L:12D per day for 6 weeks (Phase I), followed by 24L:0D per day for 6 weeks (Phase II); one group was 0 + smolt (EXP1) and the other 1 + smolt (EXP2). To monitor the smoltification status of the fish, 100 (EXP1) and 60 (EXP2) fish were randomly sampled per week during Phase II. The following phenotypes for smoltification status were studied: RT-qPCR relative mRNA expression of values of two alpha catalytic subunits of the variants of the Na^+^K^+^ATPase (NKA) expressed in the sampled gill tissues of each fish. The first variant, alpha1a with increased expression in freshwater (FW) and the second variant alpha1b with increased expression in seawater variant (SW), as well as their ratio SW/FW. At the optimal time for seawater transfer based on the SW/FW trait, 1,000 (at sixth sampling of EXP1) and 1,500 (at fifth sampling of EXP2) fish were sampled for genetic parameter estimation. The individual variation in FW, SW, and SW/FW was very large at each of the seven samplings indicating a large variation among individuals in the optimum time of transfer to seawater. SW/FW showed significant genetic variation in both 0+ and 1+ smolts, which indicates the possibility for selection for improved synchronization of smoltification status of Atlantic salmon at the time where the largest proportion of the fish is considered to be smolt. However, the genetic correlation between SW/FW of 0+ and 1+ was not significantly different from zero indicating very little shared genetic variation in SW/FW in 0+ and 1+ fish. Smoltification phenotypes showed temporal progression over the smoltification period, and this progression varied between 0+ and 1+ smolt highlighting the importance of correctly timing the major sampling point, and when cohorts are transferred to seawater. This also highlighted the need for further research into noninvasive methods of objectively measuring individual smoltification through time and subsequent smolt survival and growth rate at sea.

## Introduction

Atlantic salmon (*Salmo salar*) is the major aquaculture commodity in Norway, which in 2019 amounted to 1.357 million tons (94% of the total aquaculture production of Norway) to a value of NOK 68.1 billion [USD 7.0 billion (https://www.ssb.no)]. Mortality during grow out in the sea continues to remain a challenge for Atlantic salmon production in Norway as well as globally and represents a substantial economic loss for the farmers. Aunsmo et al. (2008) reported significant losses occurring during the first 2–3 months after seawater transfer and attributed this to suboptimal smolt production and quality, spurring intensive research into improved survival and welfare of post smolts in the seawater phase (Kolarevic et al., 2014; Ytrestøl et al., 2020).

Smoltification is the process where Atlantic salmon parr undergo behavioral, developmental, and physiological changes into smolt, which in wild fish enable their first migration downstream to the sea (Hoar, 1988) and in farmed fish to be transferred primarily into floating net cages in the sea. These changes include alterations to body shape, skin reflectance, increased sodium–potassium (Na^+^/K^+^) ATPase in gills, and the osmotic ability to regulate blood plasma ion concentrations at seawater phase (Stefansson et al., 2008). Although both wild and domesticated Atlantic salmon undergo smoltification, the timing, age, and size of the fish can be vastly different. In wild Atlantic salmon, parr are locked into smoltification once their body size and energy status reach an underlying threshold in the spring, and this can happen anytime between 1 and 8 years of age (Thorstad et al., 2012). The parr remain in the resting state until smoltification is triggered by a zeitgeber, in this case increasing photoperiod and water temperatures in the spring (Thorpe et al., 1998).

In the formative years of the Atlantic salmon industry, a population of parr would partially smoltify under natural water temperatures and photoperiods in the spring approximately 16 months after hatching and are commonly called (1+) smolts to indicate they are older than 1 year. The remaining parr, which failed to smoltify, would then continue to be cultured in freshwater until their second spring approximately 28 months after hatching and would be called (2+) smolt to indicate they are older than 2 years (Refstie et al., 1977). In the current domesticated Atlantic salmon, genetic selection for improved growth, improved feed formulation, more optimal water temperature, and rearing environments have enabled the majority of smolts to be produced at an age of 16 months after hatching (1+) and out of season smolts using artificial lighting and controlled water temperature at an age of 7–8 months after hatching. These are commonly referred to as 0+ smolts in keeping with the classification of 1+ and 2+ smolts (Mørkøre et al., 2001). An important point is that Atlantic salmon is not expressly selected for improved and synchronized 0+ or 1+ smoltification status, but rather for increased growth and there is little knowledge on whether different selection strategies are needed for these two smolt production strategies.

The physiology of seawater osmoregulation of salmonids is well understood; however, there is limited information on underlying causes of genetic variation in salinity tolerance of the fish. Currently, smoltification status of fish is determined by methods that could be classified into noninvasive and invasive. The noninvasive methods are through monitoring changes on body morphology, skin reflectance, and/or K-factor (condition factor), which tend to be less objective and accurate (Stefansson et al., 2003; Piironen et al., 2013). The invasive methods are all based on evaluating osmoregulatory ability, particularly the activity of the Na^+^/K+ ATPase enzyme (NKA) in the gills. The NKA enzyme is comprised of two essential isoforms of the alpha catalytic subunit: the alpha 1a subunit associated with expression in freshwater and ionic uptake, and the alpha 1b subunit isoform expression associated with saltwater and ionic excretion (McCormick et al., 2013). The first method includes challenging fish with exposure to full-strength saltwater and then recording the blood plasma ion concentrations (Cl^−^, Na^+^, Mg^2+^) to evaluate the performance of the NKA pathway in regulating blood ion concentrations (Stefansson et al., 2003; Piironen et al., 2013). The second method involves a gill biopsy, an enzymatic activity assay using spectrophotometry or antibodies against the NKA protein (McCormick et al., 1993; McCormick et al., 2013). Another method involves real-time quantitative polymerase chain reaction (RT-qPCR) measurements of the expression on the two isoforms of the NKA alpha subunit (McCormick et al., 2009; Handeland et al., 2014). Although the gill biopsies can be collected nonlethally (McCormick et al., 1993), in many countries including Norway, it is a legal requirement to sample fish lethally dosed with anesthesia postmortem (Stefansson et al., 2003; Piironen et al., 2013). This makes it impossible to follow the individual growth rate of fish sampled for these phenotypes after seawater transfer and also inhibits their use as breeding candidates for genetic improvement.

Phenotypes that could effectively and accurately quantify smoltification status will provide the needed information for studying the genetic architecture of smoltification. Even though several studies have reported results on mapping of quantitative trait loci (QTL) for smoltification status traits in salmonids (Boulding et al., 2008; Nichols et al., 2008; Stefansson et al., 2008; Le Bras et al., 2011), the information on genetic variation for smoltification status remains very limited. Thus, the objectives of the present study were to estimate the genetic variation for different traits related to smoltification status at the time of transfer from freshwater to seawater and the genetic correlations between these smoltification traits within and between a group of 0+ and a group of 1+ smolts partly from the same families.

## Materials and Methods

### The Environment and the Fish

The smoltification experiment was conducted at the freshwater facility of Nofima Research Station for Sustainable Aquaculture (RSSA), located at Sunndalsøra (62°40′3.5292″N, 8°31′28.974″E), Norway. In January 2017, Mowi ASA delivered around 3,000 eyed eggs of Atlantic salmon from 50 families (60 eggs per family, from 25 sires and 50 dams) to RSSA, where the eggs were hatched and reared in one tank to an average body size of 50 g, as the experimental fish (0+) for the first experiment (EXP1). In July 2017, a batch of 2,500 fingerlings of size 30 g from 150 families from 50 sires and 150 dams, which included the sibs of the 50 families that involved in EXP1, were sent from Mowi ASA to RSSA. The fingerlings were reared until they reached an average body weight of 200 g (1+) by the end of January 2018 and used in the second experiment (EXP2). All the fish were not individually identified with a PIT tag, but at the time of gill sampling and body measurement recording, a fin sample from the tail was taken for genotyping using a medium density SNP array (57K Affymetrix chip developed by Nofima) for the purpose of parentage assignment.

### The Experimental Design and Fish

The smoltification experiments were conducted in two batches with the 0+ parr from September 12 to December 5, 2017 (EXP1) and 1+ parr from February 5 to May 31, 2018 (EXP2). For EXP1, 2,000 fish (40 fish from each of the 50 families) with an average body weight of 50 g at the start of the experiment were stocked in one experimental tank of size 3 m × 3 m × 0.8 m. For EXP2, two groups of 1,000 fish each (13–14 fish from each of 150 families) with an average body weight of 200 g at the start of the experiment were stocked in two experimental tanks (T1 and T2) each of size 3 m × 3 m × 0.8 m.

**FIGURE 1:**
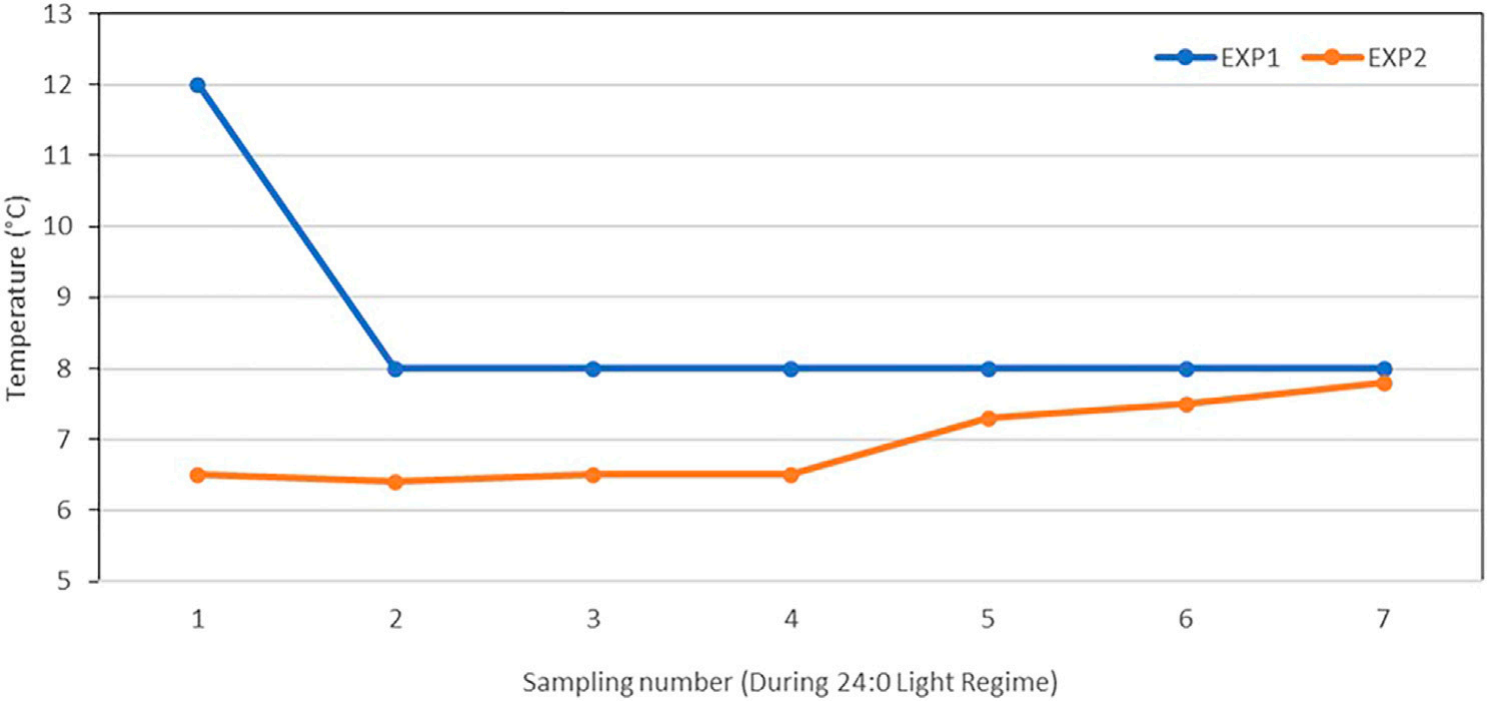
Freshwater temperatures (°C) at each of the seven samplings during Phase II of 0+ smolt (EXP1) (6 weeks) and 1+ smolt (EXP2) (10 weeks).

A standard light regime was used for smoltification, which started with alternating 12 h of light and 12 h of darkness (12L:12D) for 6 weeks (Phase I), followed by continuous (24 h, 24L:0D) light for 6 weeks (Phase II). For EXP2, Phase II lasted for 10 weeks and, thus, 4 weeks longer than planned. This was mainly due to the water temperatures at that time were lower (6.4°C–7.8°C) than during Phase I (Figure 1), and therefore, the fish needed more days to reach the desired degree-days (°d = °C × days) for smoltification [300 to 400°days after the increase in the daylength as suggested by Sigholt et al. (1998)].

For monitoring and measuring the smoltification status of the fish, 100 (EXP1) and 60 fish (EXP2; 30 fish per tank) were randomly sampled during Phase II on a weekly basis for EXP1 and at slightly longer intervals of 10–11 days for EXP2 to account for the lower temperature in EXP2 (Figure 1). At the optimal time for seawater transfer (based on the results from SmoltVision analysis provided by Pharmaq Analytiq), 1,000 (EXP1) and 1,500 fish (EXP2; 750 fish per tank) were sampled for genetic parameter estimation. For EXP1, this major sampling event took place on November 28, 2017 (at the sixth of the seven samplings), and for EXP2, on May 14, 2018 (at the fifth of the seven samplings).

The sampled fish were first anesthetized with a lethal dose of tricaine mesylate (MS-222). Once anesthetized, a gill filament was biopsied from the left side of the fish and placed in the SmoltVision tube containing RNAlater (ThermoFisher, United States) for preservation of the sample, then sent to Pharmaq Analytiq for further analysis. Immediately after gill biopsy, the fish were given a sharp blow to the head to ensure no chance of unintended recovery, as stipulated in the ethical permits. The ethical use of fish in both experiments were approved by the Norwegian Food Safety Authority (Mattilsynet) with approval IDs 13174 (EXP1) and 15310 (EXP2).

### Smoltification Phenotypes and Records

Four smoltification phenotypes were studied relating to the commercial SmoltVision test (Pharmaq Analytic, Norway): the RT-qPCR relative expression of mRNA encoding for the two protein isoforms of the NKA alpha catalytic subunit; the first one encoding for the NKA alpha 1a subunit, which is upregulated in freshwater and will be referred to as FW, and the second encoding for the NKA alpha 1b subunit, which is upregulated in saltwater and will be referred to as SW for the purpose of this study. The third phenotype is the ratio of the relative expression of the SW over FW variants referred to as SW/FW, as this is the primary quantity used in practice to determine whether fish are ready for seawater transfer. The fourth phenotype is the smolt index (SI), which is a composite phenotype calculated as the average of three subjectively scored external characteristics of the fish (parr marks, silver coloration, and fin edges, Table 1).

**TABLE 1:** Three characteristics for smolt index scoring.

Characteristic	Index (point)^a^			
Parr mark	Clear (1)	Visible (2)	Weak (3)	None (4)
Silver coloration	Clear (1)	Weak (2)	Visible (3)	Silver (4)
Fin margins	Clear (1)	Weak (2)	Visible (3)	Black margin (4)

Note:

a The transition of Atlantic salmon parr to smolt is indicated in the gradual increasing score from 1 to 4.

Importantly, the researcher must submit metadata accompanying the gill biopsy samples. This includes the tank or cage number, water temperature, light regime as well as the individual body length, body weight, and the three abovementioned external smolt characteristics (Table 1). Pharmaq Analytic returns the RT-qPCR relative expression of mRNA encoding for the two protein isoforms of the NKA alpha catalytic subunit and a second gene encoding for the NA^+^K^+^Cl⁻ co-transporter (NK1CC) used as a positive internal control, expressed relative to the elongation factor 1alpha (EF1a) housekeeping gene. Importantly, the researcher has no control over the analysis and processing of these values, and these details are proprietary information and are restricted from the user. However, the SmoltVision test is based on the methods previously described by Handeland et al. (2014).

In addition, the individual body weight (BW) and body length (BL) were recorded for each fish sampled at each of the seven samplings. The condition factor was calculated as $K-\text{factor}=\frac{\text{BW}*100}{{\text{BL}}^{b}}$ , where b = 3 as in the general formula of the K-factor (Nash et al., 2006), and K2-factor where the b was estimated for EXP1 (b = 2.845 ± 0.040) and EXP2 (b = 2.788 ± 0.019) by fitting a simple linear regression model, ln(BW)=intercept+b∗ln(BL) + *e*. This was done to obtain a K2-factor that was independent of BL of the fish (Le Cren, 1951). The phenotypic correlation of K-factor with BL in EXP1 was −0.18 (*p*-value <0.0001) and in EXP2 −0.32 (*p*-value <0.0001), while that of K2-factor with BL in EXP1 was 0.046 (*p* = 0.13) and in EXP2 0.004 (*p* = 0.88).

### Estimation of Phenotypic and Genetic Parameters

The data from EXP1 and EXP2 were first analyzed separately to obtain genetic parameters of the traits within the 1+ and 0+ smolts and, thereafter, combined primarily to obtain an estimate of the genetic correlation between the homologous trait measured in the 0+ and 1+ fish. All the parameter estimates were obtained using restricted maximum likelihood in WOMBAT (Meyer, 2007) and with the genomic relationship matrix added to the random animal effect of the mixed model equations of the applied linear mixed animal model (see below).

#### Parameter Estimates for the Different Traits Within EXP1 and EXP2

Phenotypic and genetic (co)variances for the studied traits, i.e., BW, K2-factor, FW, SW, and SW/FW ratio, were obtained from a multitrait animal model, separately for EXP1 and EXP2. In obtaining all the genetic correlations between these five traits, a three-trait (BW, K2-factor, SW/FW) and a four-trait (BW, K2-factor, FW, and SW) model was used as the parameters did not converge when all five traits were included simultaneously, specifically when combinations of SW/FW, SW, and FW were included. The four-trait model may be written as:

$$\left[\begin{array}{c} y_{1}\\ y_{2}\\ y_{3}\\ y_{4} \end{array}\right]=\left[\begin{array}{cccc} X_{1} & 0 & 0 & 0\\ 0 & X_{2} & 0 & 0\\ 0 & 0 & X_{3} & 0\\ 0 & 0 & 0 & X_{4} \end{array}\right]\left[\begin{array}{c} b_{1}\\ b_{2}\\ b_{3}\\ b_{4} \end{array}\right]+\left[\begin{array}{cccc} Z_{A_{1}} & 0 & 0 & 0\\ 0 & Z_{A_{2}} & 0 & 0\\ 0 & 0 & Z_{A_{3}} & 0\\ 0 & 0 & 0 & Z_{A_{4}} \end{array}\right]\left[\begin{array}{c} a_{1}\\ a_{2}\\ a_{3}\\ a_{4} \end{array}\right]+\left[\begin{array}{c} e_{1}\\ e_{2}\\ e_{3}\\ e_{4} \end{array}\right];$$

where for each of the traits, y is the vector of the phenotypic observations, b is the vector of fixed effect (overall mean, tank with two levels (T1 or T2) for EXP2, sex with the two levels male or female), and  X and $Z_{A}$ are the design matrices assigning observations to their levels of the fixed and animal additive genetic effects, respectively.

a is the vector of random additive genetic effects, which follows normal distribution N(0,G⊗A) , where G is the marker-based genomic relationship matrix constructed as $\frac{{\text{MM}}^{'}}{2\sum p_{i}\left(1-p_{i}\right)}$ , M is the centered marker genotypes, and $p_{i}$ is the allele frequency of each marker; A is the genetic variance covariances matrix; and e is the vector of random residual effects, which follows the normal distribution N(0,I⊗R) , where I is the identity matrix, and R is the residual variance covariances matrix.

The trait BL was not included due to its high phenotypic and genetic correlations to BW (r_g_ = 0.94 ± 0.00; r_g_ = 0.97 ± 0.01 for Exp1; r_p_ = 0.96 ± 0.00, r_g_ = 0.97 ± 0.01 for EXP2) obtained from a bivariate analysis of the data from each of the two experiments. For the trait SI in EXP2, the genetic variation was zero and was therefore not included in the model. The effect of sex was not significantly different from zero for all the traits (*p* > 0.05) and was therefore omitted from the final model.

#### Genetic Correlation Between the Homologous Trait in EXP1 and EXP2

In obtaining the genetic correlation between the six homologous traits in EXP1 and EXP2, i.e., BW, K2-factor, SI, FW, SW, and SW/FW, each trait was considered as two different traits in a bivariate model similar to the model in 2.3.1. In the bivariate model, tank effect was fitted as the fixed effect for each of the traits. For the bivariate model with the two SI traits, full convergence was not achieved. The residual correlation between the same trait in the two experiments was set equal to zero as these traits were recorded on different animals.

## Results

### Descriptive Statistics for the Traits Recorded at Each Sampling

**FIGURE 2:**
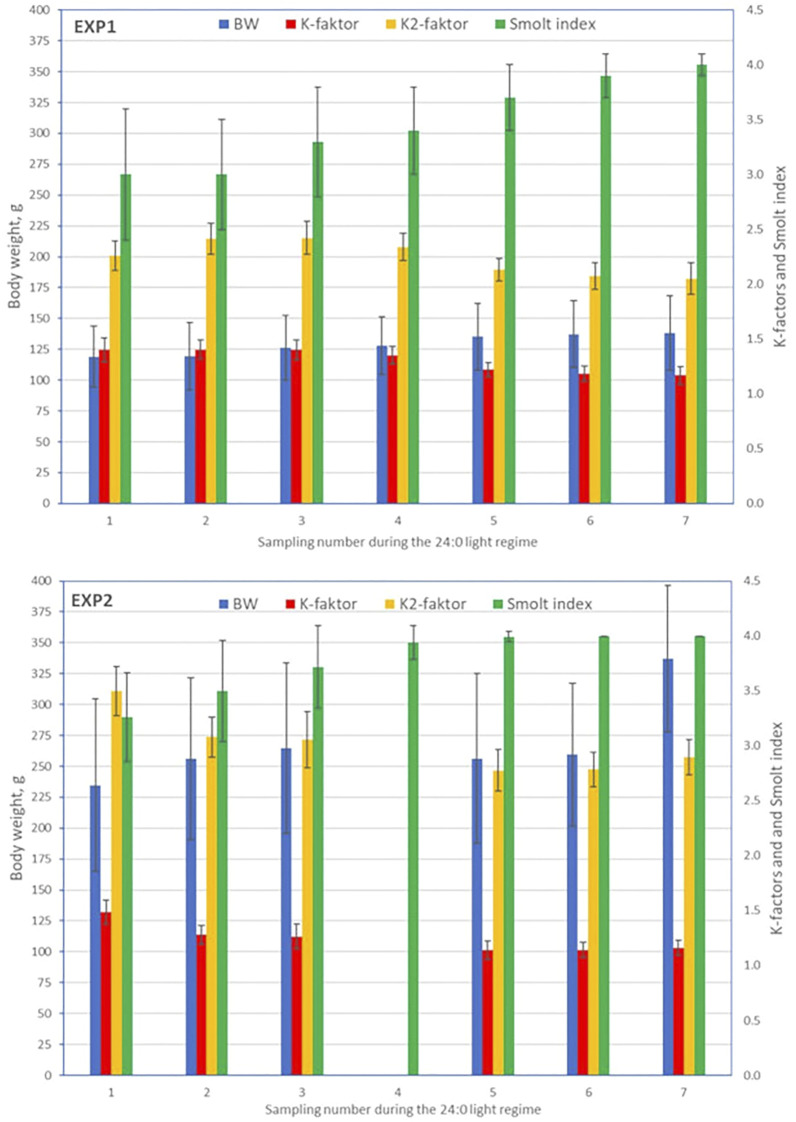
Means (±standard deviations) for body weight, K-factor, K2-factor, and smolt index at each sampling for EXP1 and EXP2.

**FIGURE 3:**
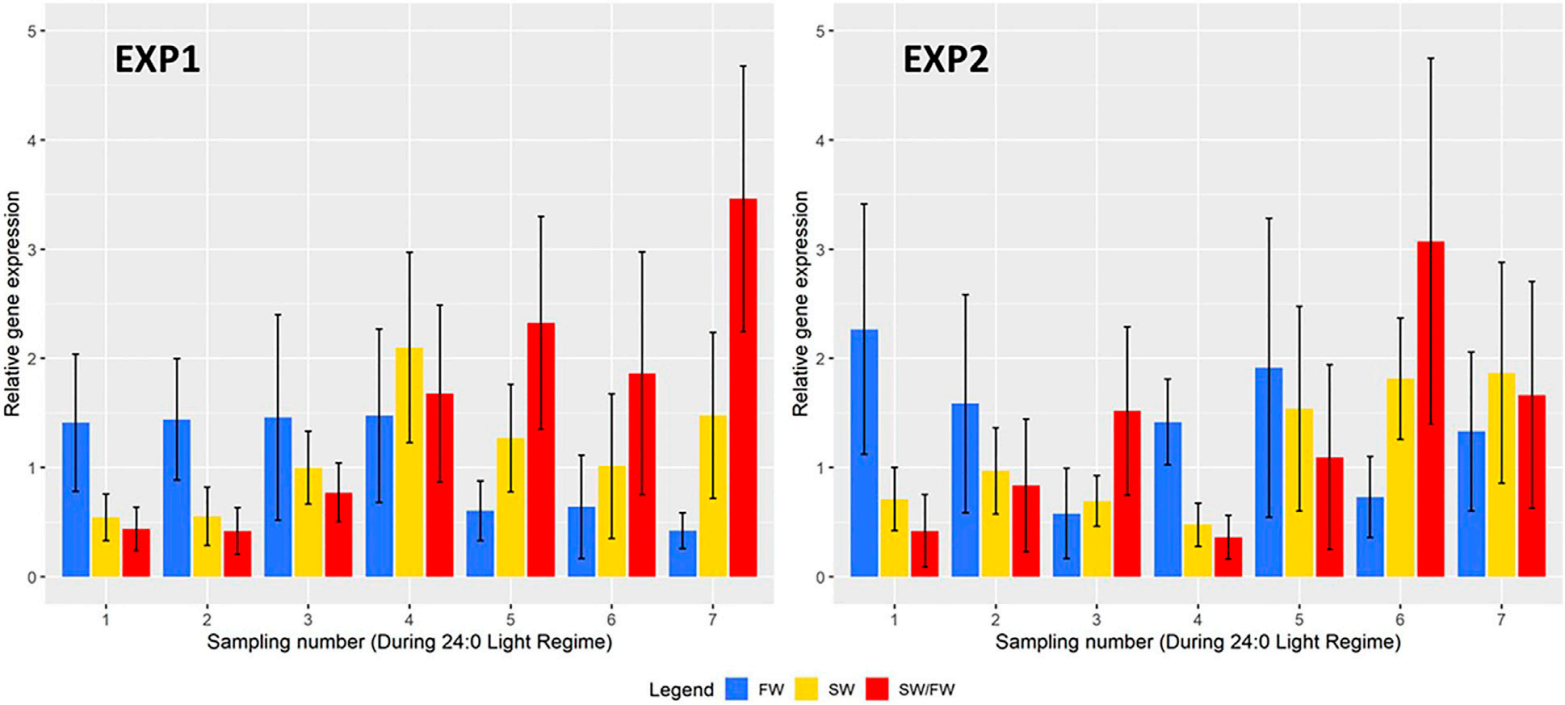
Mean real-time quantitative polymerase chain reaction (RT-qPCR) values (±their standard deviations) of two key genes {Na^+^K^+^ATPase [NKA α1a for the freshwater variant (FW); NKA α1b for the saltwater variant (SW)]} with effect on Na^+^/K^+^ATPase in the gills and their ratio (SW/FW) at each sampling for EXP1 and EXP2.

A total of 1,595 (EXP1) and 1,773 (EXP2) fish with phenotypes were recorded from the seven samplings of each of the two experiments. Separately for EXP1 and EXP2, means and standard deviations at each sampling, for BW, K-factor, K2-factor, and SI are presented in Figure 2, and for FW, SW, and SW/FW in Figure 3.

#### Body Weight, K-Factor, K2-Factor, and Smolt Index

For EXP1, mean values of BW increased over the samplings, while for EXP2, the mean BW at both fifth and sixth sampling for unknown reason were lower than at the third sampling (BW at the fourth sampling had to be omitted due to some systematic measurement error of BW and BL seen as unreasonable K-factor values). K-factor decreased from about 1.4 to 1.2 (EXP1, 16.6%) and 1.5 to 1.2 (EXP2, 21.8%), and K2-factor from about 2.3 to 2.2 (EXP1, 9.2%) and 3.5 to 2.9 (EXP2, 17.1%). Due to unknown sampling error at the fourth sampling of EXP2, the mean values for BW, K-factor and K2-factor were removed from Figure 2. The Smolt index increased gradually from 3.0 (EXP1) and 3.2 (EXP2) until its maximum value of 4.0 at the seventh sampling of EXP1 and fifth sampling of EXP2 (Figure 2).

For BW, the coefficient of variation (CV) ranged from 17 to 30% (EXP1) and 18–23% (EXP2). K-factor had a small CV ranging from 5 to 8% for EXP1 and 6–9% for EXP2, while the CV for K2-factor was marginally lower. For SI, CV decreased from 20% (EXP1) and 12% (EXP2) at the first sampling to very low values (≤5%) at the sixth and seventh sampling (EXP1) and fourth, fifth, sixth, and seventh sampling (EXP2).

#### Real-Time Quantitative Polymerase Chain Reaction Values and Their Ratio

The relative gene expression of FW, SW, and SW/FW for each sampling is shown in Figure 3 for EXP1 and EXP2. In EXP1, the mean FW was very similar (ranging from 1.41 to 1.48) at the first four samplings and decreased to a much lower value (ranging from 0.42 to 0.64) at the last three samplings. The mean SW increased gradually over the samplings and decreased to a lower value at the last three samplings. For SW/FW, the mean increased over the first five samplings, decreased to a lower value at the sixth sampling and increased to a higher mean value at the seventh sampling. At each of the seven samplings, these three traits showed a large variation among the animals as seen from their large standard deviations in Figure 3 with CV ranging from 33 to 65%.

In EXP2, the mean values of all these three traits showed large variation from one sampling to the next, most probably due to a lesser number of fish recorded per sample (30–60 compared with 100 in EXP1) and the very large CV of the traits (28–79%). For the trait SW/FW, the mean value was higher at the last two samplings than at the fifth and major sampling.

**FIGURE 4:**
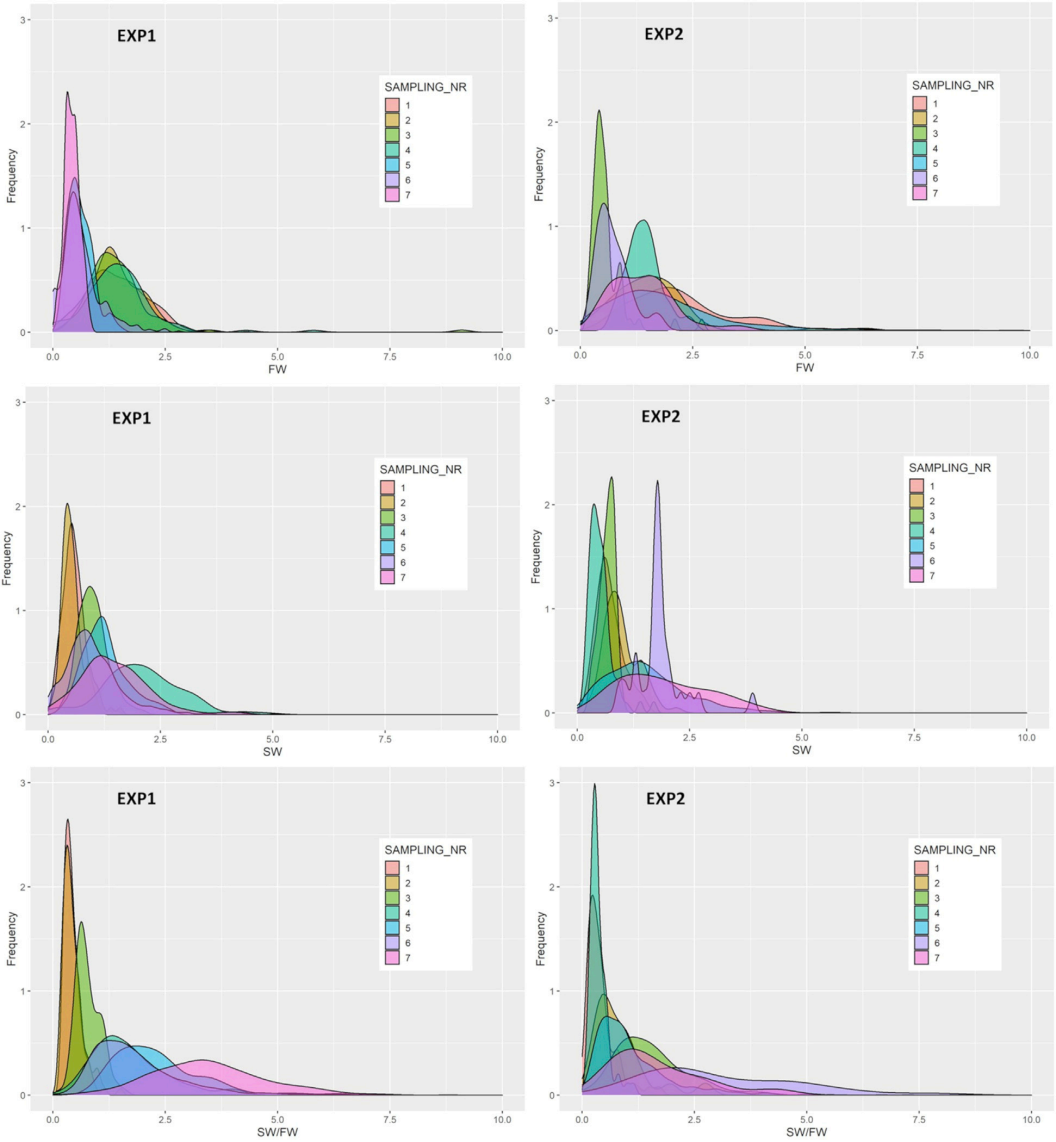
Frequency distributions of the relative expression of freshwater (FW) and the seawater (SW) variants and their ratio (SW/FW) determined by two genes with effect on the Na^+^K^+^ATPase activity in the gills at each of the seven samplings of EXP1 and EXP2.

The frequency distributions of FW, SW, and SW/FW are presented in Figure 4. For SW/FW, the variation among individuals increases over the smoltification period and were much larger during the last four compared with the three first samplings.

#### Correlations Between Traits Within Each Sampling

**FIGURE 5:**
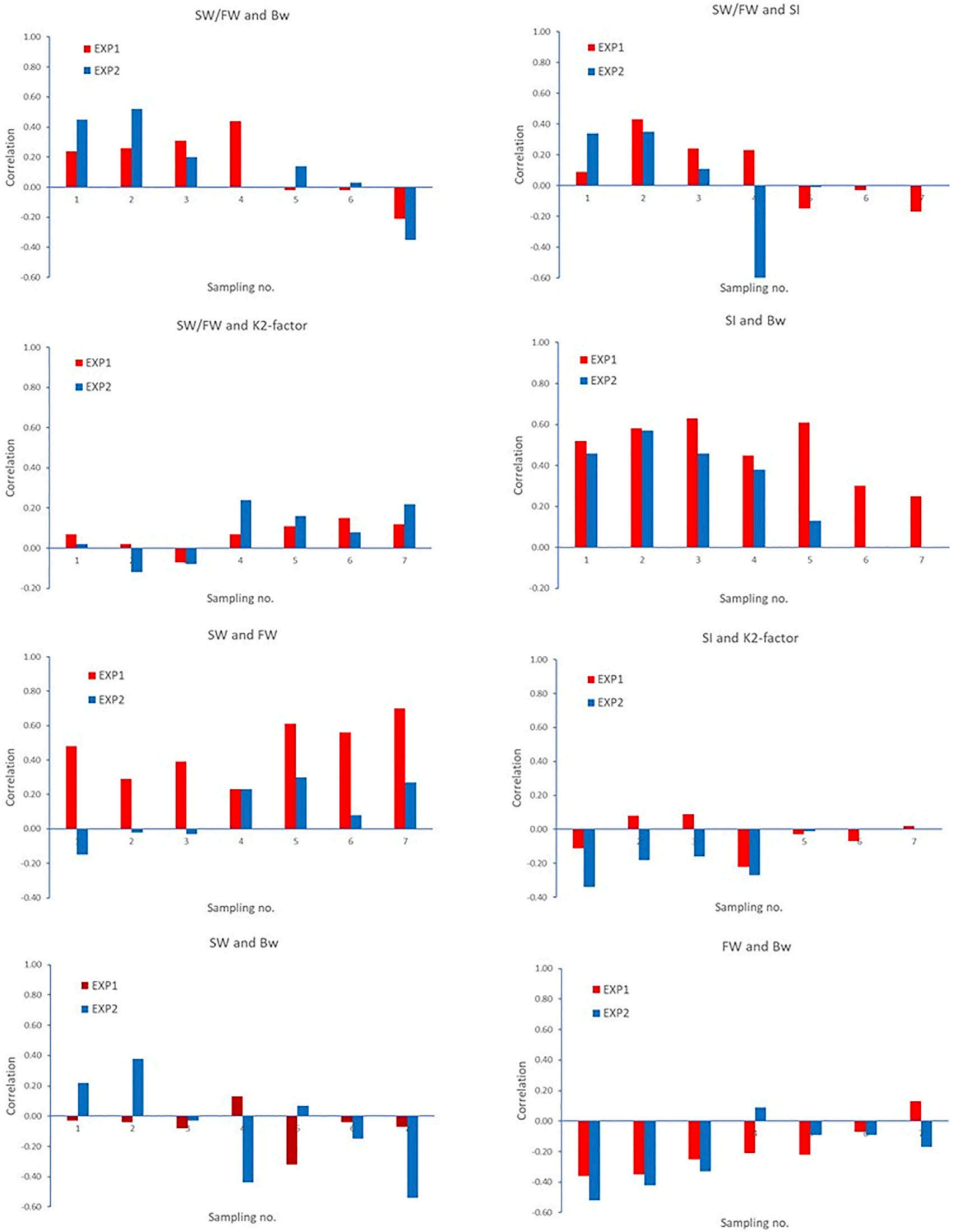
Correlations between some selected pair of traits within each of the seven samplings. Correlation −0.254 > r > 0.254 (EXP1, N = 100 fish recorded/sampling) and −0.325 > r > 0.325 (EXP2, N = 2 × 30 fish recorded/sampling) are significantly different from zero (*p* < 0.05).

Figure 5 shows the correlations between some selected pair of traits within each of the seven samplings, separately for EXP1 and EXP2. The figure shows that the correlations vary over the seven samplings and with different patterns for the two experiments.

### Descriptive Statistics for the Traits at the Major Sampling

Descriptive statistics for the traits measured at the major samplings are presented in Table 2. Average weight at this sampling was 137.7 (SD = 27.6) and 256.4 g (SD = 68.6 g) for EXP1 and EXP2, respectively. Interestingly, SI was close to the maximum mean value of 3.95 (EXP1, sixth sampling) and 3.99 (EXP2, fifth sampling) showing that, morphologically, nearly all the fish were ready for seawater transfer at the major samplings. However, based on the mean SW/FW of 1.86 (EXP1) and 1.09 (EXP2), compared with the set desired benchmark of 2.00 (personal communication PHARMAQ Analytiq AS), the fish were physiologically (on average) near (EXP1) or far from (EXP2) being ready for seawater transfer. The very large CV of SW/FW (60% for EXP1 and 78% for EXP2) indicates substantial variation among the animals with respect to their readiness for seawater transfer.

**TABLE 2:** Descriptive statistics for body weight (BW), body length (BL), condition factor (K-factor and K2-factor), freshwater variant (FW), seawater variant (SW), SW/FW ratio, and smolt index (SI) recorded at the major sampling of the fish in EXP1 (sampling 6) and EXP2 (sampling 5).

Trait	Experiment	N	Mean	Min	Max	SD	CVx100
BW	EXP1	944	137.7	11.6	215.7	27.6	20.1
	EXP2	15,00	256.4	91.1	556.0	68.6	26.8
BL	EXP1	944	22.6	13.5	26.4	1.59	7.0
	EXP2	1,500	28.4	19.8	37.0	2.62	9.2
K2-factor	EXP1	942	1.92	1.34	3.15	0.12	6.2
	EXP2	1,500	2.22	1.21	3.16	0.14	6.2
SI	EXP1	944	3.95	2.30	4.00	0.16	4.1
	EXP2	1,500	3.99	3.00	4.00	0.05	1.3
FW	EXP1	944	0.68	0.10	4.30	0.46	67.6
	EXP2	1,500	1.91	0.10	11.40	1.37	71.4
SW	EXP1	944	1.07	0.00	5.30	0.64	60.1
	EXP2	1,500	1.54	0.00	10.50	0.94	60.8
SW/FW	EXP1	944	1.86	0.00	12.00	1.11	59.6
	EXP2	1,500	1.09	0.00	7.25	0.85	77.5

#### Parameter Estimates for the Homologous Trait Between EXP1 and EXP2 at Major Sampling

In EXP1 and EXP2, similar bivariate heritability estimates were found for both BW and SW/FW, but for K2-factor, SI, FW, and SW, the heritability estimates differ by more than 50% between the experiments (Table 3). For SI, the parameter estimates did not fully converge (and the estimates were therefore unreliable), as may be expected as nearly all fish were close to the maximum possible value of 4.0 for the trait in both EXP1 and EXP2 and, thus, with very low phenotypic variation (Table 2).

**TABLE 3:** Estimates of additive genetic and residual variances and heritability from bivariate models of the same trait in EXP1 and EXP2, and the genetic (r_g_, upper line for each trait) and phenotypic (r_p_, lower line for each trait) correlation between the same trait in the two experiments.

Trait	Experiment	$\sigma_{A}^{2}$ ± s.e.	$\sigma_{e}^{2}$ ± s.e.	$h^{2}$ ± s.e.	r_g/_r_p_
BW	EXP1	543 ± 67	335 ± 28	0.62 ± 0.04	0.89 ± 0.04
	EXP2	2,918 ± 314	1,806 ± 132	0.62 ± 0.04	0.55 ± 0.04
K2-factor	EXP1	0.01 ± 0.00	0.02 ± 0.00	0.27 ± 0.04	0.92 ± 0.06
	EXP2	0.02 ± 0.00	0.01 ± 0.00	0.69 ± 0.04	0.40 ± 0.06
SI^a^	EXP1	0.003	0.023	0.12	0.82
	EXP2	0.000	0.003	0.01	0.02
FW	EXP1	0.01 ± 0.01	0.20 ± 0.01	0.04 ± 0.03	0.22 ± 0.39
	EXP2	0.19 ± 0.06	1.61 ± 0.08	0.11 ± 0.03	0.22 ± 0.39
SW	EXP1	0.02 ± 0.01	0.39 ± 0.02	0.04 ± 0.03	0.09 ± 0.38
	EXP2	0.07 ± 0.03	0.79 ± 0.04	0.09 ± 0.03	−0.01 ± 0.02
SW/FW	EXP1	0.23 ± 0.07	1.03 ± 0.06	0.18 ± 0.05	−0.11 ± 0.23
	EXP2	0.09 ± 0.03	0.62 ± 0.03	0.12 ± 0.03	−0.02 ± 0.03

a Estimates did not converge, and the estimates were, thus, not reliable.

The genetic correlation between the homologous traits in EXP1 and EXP2 were high, i.e., 0.89 ± 0.04 for BW, 0.92 ± 0.06 for K2-factor, and 0.82 for SI (Table 3). However, for the other studied traits, the genetic correlations between the homologous traits in the two experiments were close to, and not significantly different from, zero.

#### Parameter Estimates for the Different Traits Within EXP1 and EXP2

The heritability estimates from the multitrait models were similar to those obtained from the bivariate models (results not shown). Table 4 shows that the genetic correlations between BW and K2-factor was positive ranging from low to medium (0.22–0.50). Genetic correlations between BW and the smoltification traits (FW, SW, and SW/FW) were, in general, low and not significantly different from zero. However, the genetic correlation of K2-factor and the smoltification traits were low (not significantly different from zero, EXP2) to moderate (EXP1). Lastly, the genetic correlations between the two main smoltification traits (FW and SW) were low and not significantly different from zero, while the residual correlation between the two traits were positive.

**TABLE 4:** Estimates of residual (upper diagonal) and genetic (lower diagonal) correlations (±s.e.) between body measurements and smoltification status traits for EXP1 and EXP2 from a multivariate animal model.

Experiment	Trait	BW	K2-factor	FW	SW	SW/FW
EXP1	BW	—	0.26 ± 0.05	0.01	−0.01	−0.04 ± 0.05
	K2-factor	0.50 ± 0.09	—	0.04	−0.05	−0.06 ± 0.04
	FW	−0.49	−0.61	—	0.53	^a^
	SW	−0.25	0.66	−0.16	—	^a^
	SW/FW	0.07 ± 0.15	0.68 ± 0.14	^a^	^a^	—
EXP2	BW	—	0.32 ± 0.05	−0.13 ± 0.04	0.08 ± 0.04	0.14 ± 0.04
	K2-factor	0.22 ± 0.08	—	−0.13 ± 0.04	0.07 ± 0.04	0.16 ± 0.04
	FW	−0.04 ± 0.16	−0.24 ± 0.15	—	0.32 ± 0.03	^a^
	SW	0.14 ± 0.17	0.02 ± 0.16	0.03 ± 0.28	—	^a^
	SW/FW	0.16 ± 0.14	0.33 ± 0.12	^a^	^a^	—

a For EXP1, the parameter estimates did not converge for the four-trait model with BW × K2-factor × FW × SW, and the estimates are, thus, not reliable.

## Discussion

### Overall Findings

The ratio of SW/FW showed low to moderate but significant genetic variation in both 0+ and 1+ smolts in Atlantic salmon at the major sampling, which indicates the possibility for selection for improved synchronization of smoltification status at the time where the largest proportion of the fish are considered to be smolt. However, the genetic correlation between SW/FW of 0+ and 1+ was not significantly different from zero indicating very little shared genetic variation in SW/FW in 0+ and 1+ fish.

### Temporal Changes During Smoltification and Determining Seawater Transfer

Smoltification status is a temporal and reversable process, where fish can be smoltified and de-smoltified depending on the individual and the environmental conditions (Hoar, 1988; Nichols et al., 2008). In practice, controlled artificial lighting regimes are used to synchronize smoltification status in groups of fish so that the majority are ready for seawater transfer. However, we observed very different temporal progression of smoltification between the 0+ and 1+ fish in EXP1 and EXP2 as seen in Figures 2 and 3 as well as the very different patterns in the phenotypic correlations between traits across time and EXP1 and EXP2 in Figure 5.

In EXP1, the smoltification of fish showed a trend of progressive increase in SI as well as a decrease in FW and concurrent increase in SW/FW. Based on the progression of SI and SW/FW, it was predicted at sampling point five that by sampling point six, the majority of the fish were ready for seawater transfer, and this is when the major sampling occurred. This progression can be seen in Figure 5 where the phenotypic correlations between SW/FW and SI were moderate and positive until sampling five at which point they changed sign to moderate negative. Crucially, based on SW/FW at the major sampling of EXP1, 347 (with SW/FW ratio value of ≥2) of 944 fish (36.8%) would have been deemed ready for transfer to the sea, while based on SI, 924 (with a Smolt index value of ≥3.5) of 944 (97.9%) were deemed ready for transfer to the sea.

However, EXP2 showed differences between smoltification phenotypes through time and differences compared with EXP1. A steady increase in SI was observed in the first four samplings, which then remained stable until sampling seven. While FW steadily decreased, SW increased, and consequently, SW/FW also increased over the first three samplings, followed by an abrupt change at sampling four. This progress is very clear in Figure 5 where the phenotypic correlations between SI and SW/FW were moderate and positive but suddenly changed sign to strongly negative at sampling point four. At this point in time, it was not clear if the changes at sampling four were due to random sampling error in the 60 fish or if the optimal time of seawater challenge might have been missed and seawater transfer (major sampling) should occur at point five. However, when all samplings in EXP2 are viewed, it appears that there was a progression toward smoltification in the first three samplings followed by asynchronous smoltification at sampling point four, although it is also possible there was de-smoltification at sampling four and then subsequent re-smoltification at sampling six and seven. These changes could also reflect stochastic differences due to sampling sizes of 60 fish in the seawater challenges for EXP2. However, the major sampling at point 5 of 1,500 fish is consistent with the temporal trend with the smaller sampling on 60 fish and, thus, supports the notion that we observed asynchronous smoltification in EXP2 and not differences due to sampling size. Importantly, only 602 (40.1%) out of 1,500 fish were deemed ready for seawater transfer at major sampling based on their individual SW/FW, while based on SI, almost all (99.7%) 1.500 (with a Smolt index value of ≥3.5) were ready for seawater transfer. Collectively, these findings suggest that there is discordance in predicting smoltification status by SI and SW/FW in both 0+ and 1+ smolt.

An important consideration is that the body size profile of 1+ Atlantic salmon smolt is not static. In the mid-1980s, 1+ smolt were typically 30–50 g at seawater transfer, and this increased to 70–120 g by the early 2000s (Bergheim et al., 2009) to 150–250 g at present (personal communication, Mowi ASA). Research report large losses of smolt 2–3 months post seawater transfer (Aunsmo et al., 2008) and that larger 0+ smolt appears to have better hypo-osmoregulatory ability (Handeland and Stefansson et al., 2001). In addition, evidence that the full switch to RAS (recirculating aquaculture system) production of smolts in the Faroe Islands after the year 2000 and the subsequent larger smolts resulted in a reduction in sea cage mortality rate (Joensen, 2008, Bergheim et al., 2009). This has led to considerable research interest in producing larger 450 to 1,000 g of 1+ smolts in land-based facilities, in attempts to improve survival and growth after seawater transfer (Ytrestøyl et al., 2020; Bergheim, 2012; Kolarevic et al., 2014). In contradiction, the present study found inconsistencies between different smoltification parameters in 0+ and the larger 1+ smolts during smoltification with evidence of possible asynchronous or de-smoltification in larger 1+ smolts. Suggesting that accurate prediction of smoltification status and synchronization of smoltification may be more challenging in larger smolts. Furthermore, SI and SW/FW gave contradictory indications of smoltification status in both 0+ and 1+ smolts, and given the wide array of smoltification regimes and smoltification phenotypes used in Atlantic salmon production, we speculate that contributing factors to the smolt mortality in the first 3 months after transfer to seawater may be due to incorrect determination of smoltification status and asynchronous smoltification status. This agrees with the findings of Kristensen et al. (2012) who surveyed mortality in the first 90 days post seawater transfer in commercial production in Norway and found increased mortality in 1+ compared with 0+ smolt despite a trend in increasing size of 1+ smolt. A more recent analysis of mortality in the Norwegian Atlantic salmon industry further confirmed higher mortalities in 1+ compared with 0+ smolts as well as an adverse effect of increasing body weight of smolts at seawater transfer (Oliveira et al., 2021).

### Genetic Variation for Smoltification Status traits—Seawater Variant/Freshwater Variant and Smolt Index

The smolt index and SW/FW were the two primary traits used in this study for quantifying the smoltification status of the 0+ and 1+ fish subjected to a controlled artificial lighting regime. The differences between the 0+ and 1+ groups described above persisted at the genetic level. For example, SI had significant genetic variation for 0+ fish (h^2^ = 0.12), whereas for the 1+ fish, SI had practically zero phenotypic and genetic variation. For the SW/FW, both 0+ and 1+ populations had moderate heritability, indicating significant additive genetic control on gill Na^+^K^+^ATPase activity, which could be used toward genetic improvement on readiness of smolts for seawater transfer. However, SW and FW had low nonsignificant heritability in EXP1 (h^2^ = 0.04) but did have a low but significant heritability in EXP2 (h^2^ = 0.09–0.11). To the best of our knowledge, there have been no similar studies on quantifying smoltification status by using SW/FW or SI on Atlantic salmon or any other salmonids. There are studies that categorized smoltification into a binary trait, which makes direct comparisons challenging but still provide evidence for genetic variation in smoltification status. The first of which was by Refstie et al (1977), who defined 1+ smolts as fish that exceed a particular size threshold and found this to be heritable (0.16 ± 0.05) in Atlantic salmon. The second study used a similar smoltification index to define a binary smoltification trait for captive 1+ Atlantic salmon smolts derived from wild fish two generations previously and found h^2^ of 0.60 and 0.48 for fish reared on two temperature regimes of 2°C apart (Debes et al., 2020).

Interestingly, the SW/FW for 0+ and 1+ were not genetically correlated, and this has implications on how SW/FW could be incorporated in selective breeding schemes as it indicates these are genetically two distinct traits. Another consideration is that SW/FW ratio as it is used here is aimed at breeding for fish that shows better synchronization in smoltification status given their size and a standardized smoltification regime. As such, it may be that the genetic background to synchronizing smoltification could be different between 0+ and 1+ smolts given their size and age at the onset of a smoltification regime. The differences in the genetic background of SI and SW/FW traits between 0+ and 1+ could also be that SI does not reflect a change after the first smoltification and reflects a permanent phenotypic change on the time scale the fish were studied on, while the changes in expression in SW/FW could be more reactive and dynamic measurements, which can detect de-smoltification over short time spans. This trial made use of flow-through land-based systems with ambient water temperatures, which were different between EXP1 and EXP2 (Figure 1). It could be that different age groups may react differently to the environmental cues for smoltification, such as water temperatures and light regimes. For example, McCormick et al. (2000), found that the increase in gill Na^+^K^+^ATPase and decrease in K-factor was more advanced for fish reared in 10°C compared with those reared in 2°C water, when both were subjected to the same daylength. Similarly, Debes et al. (2020) report differences in the heritability estimates for a binary smoltification trait between fish cultured on water temperature regimes 2°C apart. This may explain the difference in terms of smolt window for 0+ and 1+ fish in our two experiments. More research on the genetic background of synchronized smoltification under different water temperatures, smolt sizes, and light regimes are certainly needed.

### The Genetic Relationships Between Body Size and Smoltification Status

The earliest research into smoltification revealed a bimodal growth of Atlantic salmon parr with the larger parr smoltifying as 1+ and the smaller smoltifying as 2+ (Kristinsson et al., 1985). This phenomenon was linked to a body size threshold for smoltification (Elson., 1957; Wedemeyer et al., 1980). With developments in Atlantic salmon husbandry including improved nutrition and genetics, this body size was reached at an earlier age as 1+, and during the last 10–15 years also as 0+ smolt (Mørkøre and Rørvik, 2001). In the first generations of captive Atlantic salmon breeding, strong genetic relationships were observed between body weight and smoltification status (binary) (r_g_ 0.89–0.85; Refstie et al., 1977). In a recent study where F2 unselected offspring of wild-caught Atlantic salmon were reared under modern rearing conditions, an equally strong genetic correlation of 0.92 was found between body length and smoltification status of 1+ smolts (Debes et al., 2020). These findings suggest that selection for improved growth rate and, thus, increased size should result in improved smoltification. The study of Refstie et al (1977) and Debes et al. (2020) both reported smolt percentages in the range of 18–78%. However, based on SI, in the present study, smolt percentages were in the range of 98% for 0+ to 99.7% 1+. Despite finding large genetic variation for body size (h^2^ = 0.62) in both 0+ and 1+ smolts, the estimated genetic correlations between body weight and smoltification status defined by SW/FW was weak (0.07 and 0.16) and not significantly different from zero. This suggests that the more recent and further selection for increased growth rate will not have improved overall smoltification rates, but instead, it has shortened the time needed for parr to reach the critical size threshold for smoltification. This suggests a genetic uncoupling of size and smoltification and would suggest that novel phenotypes more directly related to hypo-osmoregulation, like SW/FW, are needed to improve smolt quality.

In previous salmonid smoltification studies, K-factor is one of the traits commonly used as an indicator of smoltification status (Hoar, 1988; Sigholt et al., 1998; McCormick et al., 2000; Nichols et al., 2008). In general, fish that are ready for seawater transfer will have a reduced K-factor relative to contemporaries that are not ready, i.e., fish are growing relatively more in length than in weight from parr to smolt, toward a more streamlined body shape (Folmar and Dickhoff, 1980; Hoar, 1988; McCormick et al., 2000; Nichols et al., 2008). Selection for reduced K-factor as a means for improving the synchronization of smoltification status is not recommended as reduced K-factor goes against the primary breeding goal of Atlantic salmon for increased size and growth. However, genetic variation for the time of smoltification should result in significant heritability for both K2-factor and SW/FW as found in this study. As K2-factor is expected to decrease (as found in both EXP1 and EXP2) and SW/FW is expected to increase (as found in EXP1, but variable in EXP2) toward the optimum time for sea transfer, the correlation between K2-factor and SW/FW is expected to be negative. However, in this study, the genetic correlation between these two traits was positive, and the residual correlation is close to zero. Furthermore, in both EXP1 and EXP2, the phenotypic correlations between these traits were, in general, low negative in the first three samplings but changed to positive in the last four samplings. These temporal changes in traits highlight one of the greatest challenges in smoltification research, i.e., lack of knowledge and understanding of the changes in phenotypes of individual fish during the smoltification transformation due to lack of data from repeated samplings of individuals. In addition, it highlights the importance of selecting the correct time point for phenotyping large numbers of fish needed for genetic evaluation studies, as sampling too early or too late can result in low phenotypic variation as seen for SI in EXP2. Furthermore, sampling at different times can have large implications for the genetic relationships between smoltification status and K2-factor.

Last, studies are needed to genetically link smoltification status traits like SW/FW with growth and survival of smolt at sea, particularly during the first weeks after seawater transfer, which are the ultimate breeding objectives of smoltification status. A limitation of the SW/FW phenotype is that it remains difficult to measure, as gill biopsy sampling must be conducted postmortem in some countries including Norway. This is due to limited information on the health and welfare of smolt post gill biopsy for phenotypes like SW/FW, upon which animal ethical licenses can be applied for and assessed. McCormik et al. (1993) demonstrated a noninvasive procedure for fill biopsy in Altantic salmon from 44 to 77 g with no resultant mortality and no significant effect on growth and salinity tolerance after 26 days. At present, the Norwegian Food Safety Authority Mattilsynet, (https://www.mattilsynet.no/) has granted animal ethic approval for two studies investigating growth and survival of post smolts at sea after gill biopsy for smoltification phenotypes. This avenue of future research may better define the use of smoltification phenotypes from live or postmortem gill biopsies.

## Conclusions

The smoltification phenotype SW/FW showed low to moderate, but significant, genetic variation in both 0+ and 1+ smolts indicating that this could be used in selective breeding to improve synchronization of smoltification. However, the low genetic correlations between SW/FW in 0+ and 1+ fish indicate that these traits have a different genetic background depending on the size and age of the smolt. Furthermore, smoltification phenotypes showed temporal progression over the smoltification period, and this progression varied between 0+ and 1+ smolt highlighting the importance of correctly timing the point at which phenotypes are measured and cohorts are transferred to seawater. In addition, this also highlighted the need for further research into noninvasive methods of objectively measuring individual smoltification and subsequent survival and growth at sea.

## Data Availability

The original contributions presented in the study are included in the article/Supplementary Material, further inquiries can be directed to the corresponding author.
