# Supplementary material for: Quantitative Genetics of Smoltification Status at the Time of Seawater Transfer in Atlantic Salmon (Salmo Salar)
Source: Front Genet. 2021 Nov 1;12:696893. doi: 10.3389/fgene.2021.696893 (PMC8591024; doi:10.3389/fgene.2021.696893)
Supplement: Supplementary file 1 [file Table1.DOCX]

Supplementary Material

# Supplementary Tables

Supplementary Table 1. Correlations -0.254 > r > 0.254 (EXP1, N=100 fish recorded/sampling) and -0.325 > r > 0.325 (EXP2, N=60 fish recorded/sampling) are significantly different from zero (P<0.05).

| Trait | Sampling no. | K2-factor | | SI | | FW | | SW | | SW/FW | |
| --- | --- | --- | --- | --- | --- | --- | --- | --- | --- | --- | --- |
|  |  | EXP1 | EXP2 | EXP1 | EXP2 | EXP1 | EXP2 | EXP1 | EXP2 | EXP1 | EXP2 |
| BW | 1 | 0.27 | 0.10 | 0.52 | 0.46 | -0.36 | -0.52 | -0.03 | 0.22 | 0.24 | 0.45 |
|  | 2 | 0.16 | 0.07 | 0.58 | 0.57 | -0.35 | -0.42 | -0.04 | 0.38 | 0.26 | 0.52 |
|  | 3 | 0.24 | 0.17 | 0.63 | 0.46 | -0.25 | -0.33 | -0.08 | -0.03 | 0.31 | 0.20 |
|  | 4 | 0.22 | -0.69 | 0.45 | 0.38 | -0.21 | 0.09 | 0.13 | -0.44 | 0.44 | -0.45 |
|  | 5 | 0.06 | 0.19 | 0.61 | 0.13 | -0.22 | -0.09 | -0.32 | 0.07 | -0.02 | 0.14 |
|  | 6 | 0.24 | 0.17 | 0.30 | NA | -0.07 | -0.09 | -0.04 | -0.15 | -0.02 | 0.03 |
|  | 7 | 0.37 | 0.02 | 0.25 | NA | 0.13 | -0.17 | -0.07 | -0.54 | -0.21 | -0.35 |
| K2-factor | 1 | - | - | -0.11 | -0.34 | -0.23 | 0.07 | -0.02 | -0.040 | 0.07 | 0.02 |
|  | 2 | - | - | 0.08 | -0.18 | -0.05 | 0.09 | -0.01 | -0.11 | 0.02 | -0.12 |
|  | 3 | - | - | 0.09 | -0.16 | 0.10 | 0.04 | 0.03 | 0.16 | -0.07 | -0.08 |
|  | 4 | - | - | -0.22 | -0.27 | -0.10 | 0.13 | 0.08 | 0.34 | 0.07 | 0.24 |
|  | 5 | - | - | -0.03 | -0.01 | -0.07 | -0.08 | 0.08 | 0.08 | 0.11 | 0.16 |
|  | 6 | - | - | -0.07 | NA | -0.09 | -0.14 | 0.05 | -0.17 | 0.15 | 0.08 |
|  | 7 | - | - | 0.02 | NA | 0.01 | -0.41 | 0.05 | -0.06 | 0.12 | 0.22 |
| SI | 1 | - | - | - | - | -0.09 | -0.43 | 0.05 | 0.27 | 0.09 | 0.34 |
|  | 2 | - | - | - | - | -0.29 | -0.32 | 0.18 | 0.25 | 0.43 | 0.35 |
|  | 3 | - | - | - | - | -0.16 | 0.05 | 0.00 | 0.04 | 0.24 | 0.11 |
|  | 4 | - | - | - | - | -0.03 | 0.11 | 0.13 | -0.56 | 0.23 | -0.60 |
|  | 5 | - | - | - | - | -0.02 | 0.03 | -0.23 | 0.02 | -0.15 | -0.01 |
|  | 6 | - | - | - | - | -0.04 | NA | 0.01 | NA | -0.03 | NA |
|  | 7 | - | - | - | - | -0.07 | NA | -0.22 | NA | -0.17 | NA |
| FW | 1 | - | - | - | - | - | - | 0.48 | -0.15 | -0.60 | -0.64 |
|  | 2 | - | - | - | - | - | - | 0.29 | -0.02 | -0.42 | -0.60 |
|  | 3 | - | - | - | - | - | - | 0.39 | -0.03 | -0.51 | -0.60 |
|  | 4 | - | - | - | - | - | - | 0.23 | 0.23 | -0.63 | -0.37 |
|  | 5 | - | - | - | - | - | - | 0.61 | 0.30 | -0.52 | -0.48 |
|  | 6 | - | - | - | - | - | - | 0.56 | 0.08 | -0.39 | -0.72 |
|  | 7 | - | - | - | - | - | - | 0.70 | 0.27 | 0.07 | -0.50 |
| SW | 1 | - | - | - | - | - | - | - | - | 0.27 | 0.63 |
|  | 2 | - | - | - | - | - | - | - | - | 0.67 | 0.41 |
|  | 3 | - | - | - | - | - | - | - | - | 0.23 | 0.50 |
|  | 4 | - | - | - | - | - | - | - | - | 0.38 | 0.80 |
|  | 5 | - | - | - | - | - | - | - | - | 0.28 | 0.43 |
|  | 6 | - | - | - | - | - | - | - | - | 0.37 | 0.55 |
|  | 7 | - | - | - | - | - | - | - | - | 0.71 | 0.62 |
